# Strong Enrichment of Aromatic Residues in Binding Sites from a Charge-neutralized Hyperthermostable Sso7d Scaffold Library*

**DOI:** 10.1074/jbc.M116.741314

**Published:** 2016-08-30

**Authors:** Michael W. Traxlmayr, Jonathan D. Kiefer, Raja R. Srinivas, Elisabeth Lobner, Alison W. Tisdale, Naveen K. Mehta, Nicole J. Yang, Bruce Tidor, K. Dane Wittrup

**Affiliations:** From the ‡ Koch Institute for Integrative Cancer Research and; Departments of ¶ Biological Engineering and; § Chemical Engineering, Massachusetts Institute of Technology, Cambridge, Massachusetts 02139 and; ‖ Department of Chemistry, Division of Biochemistry, BOKU-University of Natural Resources and Life Sciences, 1190 Vienna, Austria

**Keywords:** directed evolution, epidermal growth factor receptor (EGFR), molecular evolution, protein stability, scaffold protein, aromatic amino acids, protein charge, yeast display

## Abstract

The Sso7d protein from the hyperthermophilic archaeon *Sulfolobus solfataricus* is an attractive binding scaffold because of its small size (7 kDa), high thermal stability (*T_m_* of 98 °C), and absence of cysteines and glycosylation sites. However, as a DNA-binding protein, Sso7d is highly positively charged, introducing a strong specificity constraint for binding epitopes and leading to nonspecific interaction with mammalian cell membranes. In the present study, we report charge-neutralized variants of Sso7d that maintain high thermal stability. Yeast-displayed libraries that were based on this reduced charge Sso7d (rcSso7d) scaffold yielded binders with low nanomolar affinities against mouse serum albumin and several epitopes on human epidermal growth factor receptor. Importantly, starting from a charge-neutralized scaffold facilitated evolutionary adaptation of binders to differentially charged epitopes on mouse serum albumin and human epidermal growth factor receptor, respectively. Interestingly, the distribution of amino acids in the small and rigid binding surface of enriched rcSso7d-based binders is very different from that generally found in more flexible antibody complementarity-determining region loops but resembles the composition of antibody-binding energetic hot spots. Particularly striking was a strong enrichment of the aromatic residues Trp, Tyr, and Phe in rcSso7d-based binders. This suggests that the rigidity and small size of this scaffold determines the unusual amino acid composition of its binding sites, mimicking the energetic core of antibody paratopes. Despite the high frequency of aromatic residues, these rcSso7d-based binders are highly expressed, thermostable, and monomeric, suggesting that the hyperstability of the starting scaffold and the rigidness of the binding surface confer a high tolerance to mutation.

## Introduction

Antibodies are broadly used as research reagents and therapeutics because of their ability to recognize a wide range of target molecules with high affinity and specificity. However, applications requiring cytoplasmic expression, small size, complex modular fusion protein topologies, or high stability motivate exploration of alternative protein scaffolds for molecular recognition.

The ideal general binder scaffold would exhibit properties such as small size, lack of glycosylation sites and disulfide bonds, high stability, high solubility, negligible aggregation, and absence of polyspecificity. A number of alternative scaffolds have been developed by diversifying the amino acid composition at the surface of a stable structure (1). Examples include designed ankyrin repeat proteins (2–4), anticalins (5, 6), affibodies (7), OBodies (8), and cystine-knot miniproteins (knottins) (9–11) among others.

**FIGURE 1.**
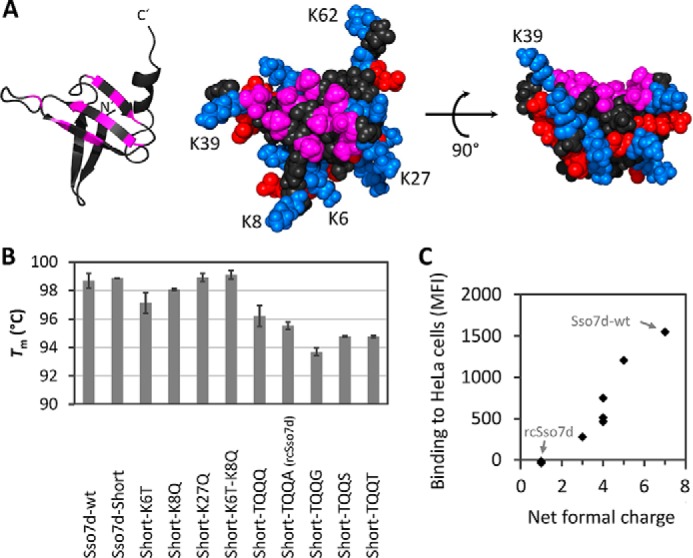
**Charge neutralization of the positively charged Sso7d scaffold.** *A*, structure of Sso7d determined by solution NMR (Protein Data Bank code 1SSO (41)). The structures on the *left* and in the *middle* are shown in the same orientation, whereas the structure on the *right* is rotated by 90°. Positions that were randomly mutated in the libraries are depicted in *magenta*. The side chains at these randomized positions were mutated to alanines in this structure by using PyMOL to better display the base of the binding surface. In the space-filling models, the positively charged residues Arg and Lys are shown in *blue*, and the negatively charged side chains of Asp and Glu are colored in *red*. The C-terminal amino acid Lys-63 is not resolved in this structure. This figure was prepared using PyMOL. *B*, *T_m_* values of partially neutralized Sso7d mutants measured by DSC (*error bars* represent S.D. of two to four measurements). *C*, Sso7d mutants with various net formal charges were tested for binding to HeLa cells. Sso7d mutants were expressed as His_6_-SUMO fusions, incubated with HeLa cells at a concentration of 1 μm, and subsequently detected with an anti-His-APC antibody. Averages of three independent experiments are shown. Sso7d-WT and the mutant rcSso7d are highlighted. *MFI*, mean fluorescence intensity.

Another recently introduced class of binder scaffolds are the homologous proteins Sso7d (12, 13) and Sac7d (14, 15) from the hyperthermophilic archaea *Sulfolobus solfataricus* and *Sulfolobus acidocaldarius*, respectively. Several advantages make these proteins particularly promising as binder scaffolds. (i) They are highly stable with *T_m_* values of 98 and 91 °C for Sso7d and Sac7d, respectively (16, 17). (ii) They are small (7 kDa) single domain proteins. (iii) They lack cysteines and glycosylation sites. Although the absence of disulfide bonds and glycosylation sites enables bacterial expression and intracellular applications, the exceptionally high stability offers additional advantages for the protein engineering process. Highly stable proteins have been shown to be more evolvable because they tolerate a wider range of mutations without losing their native fold (18). Moreover, the engineered binding site on Sso7d and Sac7d is located on the surface of a rigid β-sheet (Fig. 1*A*) (12, 14) as opposed to that of antibodies and other alternative scaffolds such as fibronectin type III (Fn3)^3^ domains, which are composed of flexible loops. Such a rigid paratope is expected to reduce the entropic penalty upon binding. Finally, stable single domain proteins are expected to lead to better expression and solubility when fused to other proteins such as antibodies or cytokines.

In the present study, we further improved the Sso7d scaffold, which is the more stable homolog (98 *versus* 91 °C for Sac7d) (16, 17). Apart from the beneficial properties discussed above, there are also drawbacks associated with Sso7d. Because Sso7d (like Sac7d) is a DNA-binding protein, it is highly positively charged with 14 of 63 residues (22%) being lysines. Basic proteins are undesirable because they bind nonspecifically to anionic mammalian cell surfaces (19–21). Moreover, the binding surface on Sso7d is surrounded by a ring of positive charges (Fig. 1*A*), which could strongly inhibit interaction with positively charged antigens or epitopes (22).

Therefore, we constructed a charge-neutralized Sso7d mutant that largely maintains high thermal stability. We demonstrate that this charge neutralization reduces nonspecific interactions with mammalian cells. Based on the neutralized scaffold, high affinity binders against mouse serum albumin (MSA) and human epidermal growth factor receptor (hEGFR) were generated. Surprisingly, the distribution of amino acids on the rigid binding surfaces of Sso7d-based binders is very different from that of the flexible complementarity-determining region (CDR) loops of antibodies but closely matches that of antibody binding energetic hot spots. In particular, the aromatic residues Trp, Tyr, and Phe were observed at high frequencies. Remarkably, binders tolerated up to four of these aromatic residues in their nine-residue binding sites and maintained high thermal stability and monomeric behavior without any observed aggregation. Together, these data demonstrate that the optimal distribution of amino acids in small, rigid binding surfaces is distinct from the composition of loop-based paratopes and that the hyperstability of the scaffold promotes an unusually high tolerance for aromatic residues.

## Results

### 

#### 

##### Charge Neutralization of Sso7d

First, we aimed at neutralizing the charge of Sso7d without significantly impacting its thermal stability. We started by deleting the two C-terminal lysines (Lys-62 and Lys-63). Differential scanning calorimetry (DSC) analysis demonstrated that the *T_m_* of the resulting mutant Sso7d-Short was not decreased (Fig. 1*B*). Next, lysines at positions Lys-6, Lys-8, Lys-27, and Lys-39 were mutated to Leu, Ile, Asn, Gln, Ser, or Thr *in silico*. These residues were chosen based on both their net charge and size. The most stable *in silico* mutation at each position was subsequently incorporated into Sso7d-Short and analyzed by DSC (Fig. 1*B*). Compared with wild-type Sso7d (Sso7d-WT; *T_m_* of 98.7 °C), the *T_m_* value of the mutant Short-K27Q was unchanged (98.9 °C), whereas the mutants Short-K6T and Short-K8Q were slightly destabilized by 1.6 and 0.6 °C, respectively (Fig. 1*B*). *In silico* calculations predicted that mutating both residues (K6T and K8Q) simultaneously would result in improved stability, whereas individual mutations would lead to destabilization. Consistent with *in silico* results, combining these mutations completely restored thermal stability, resulting in a *T_m_* of 99.1 °C. Thus, we identified the neutralizing mutations K6T, K8Q, and K27Q, which do not affect thermal stability.

At position Lys-39, *in silico* experiments identified a favorable mutation to either Gln or Thr. Because this residue protrudes from the anticipated binding surface (Fig. 1*A*, structure on the *right*), potentially sterically clashing with bound antigen, we tested mutations not only to Gln and Thr but also to the small side chains of Ala, Gly, and Ser. These mutations at position 39 were combined with the above mentioned mutations (K6T, K8Q, K27Q, and the C-terminal truncation), resulting in five clones termed Short-TQQ*X* with *X* being either Gln, Ala, Gly, Ser, or Thr at position 39. Short-TQQQ was the most stable version followed by Short-TQQA (Fig. 1*B*). Given the small side chain of alanine at position 39, which reduces the risk of clashing with the antigen, we chose Short-TQQA as our final scaffold. This mutant was termed reduced charge Sso7d (rcSso7d). Importantly, the net formal charge of rcSso7d was only +1 as opposed to +7 for Sso7d-WT, and the protein remained highly stable (*T_m_* of 95.5 °C).

To test whether this charge neutralization had an effect on nonspecific cell binding, we analyzed the binding of all mutants to HeLa cells. Remarkably, nonspecific binding strongly correlated with the net formal charge of the proteins (Fig. 1*C*) with the final scaffold rcSso7d showing no binding.

##### Construction of Libraries with Tailored Diversity

**FIGURE 2.**
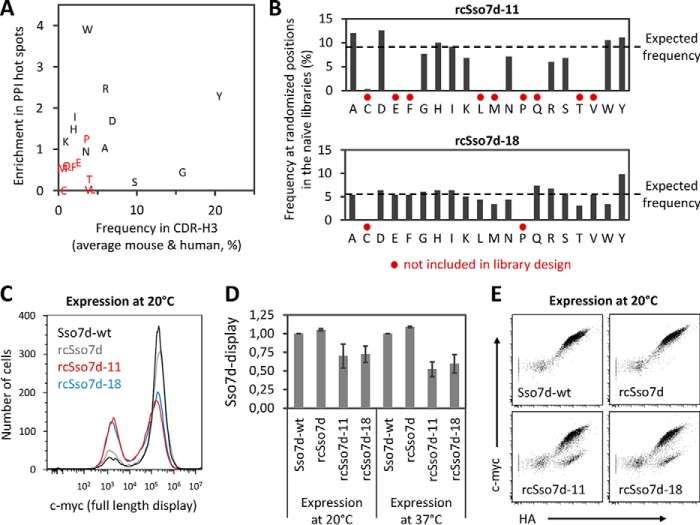
**Construction and analysis of rcSso7d libraries.** *A*, enrichments of all amino acids in PPI hot spots (23) are plotted against their frequencies in CDR-H3 loops of murine and human antibodies (24). Only the amino acids in *black* were included in the rcSso7d-11 library, whereas the rcSso7d-18 library contained all amino acids except for Cys and Pro. *B*, amino acid distributions at the randomly mutated binding site positions were analyzed by sequencing 39 and 33 randomly picked clones from the naïve libraries rcSso7d-11 and rcSso7d-18, respectively. Amino acids that were not included in the library design are marked with *red dots*, and the expected frequencies are indicated by a *dashed line. C* and *D*, Sso7d-WT, rcSso7d, and the rcSso7d-11 and rcSso7d-18 libraries were expressed on the surface of yeast at 20 or 37 °C followed by detection of full-length Sso7d mutants by using mouse anti-c-MYC. In *C*, one representative experiment of three is shown (expression at 20 °C). In *D*, expression levels on c-MYC-positive cells after normalization for Sso7d-WT expression are shown for both 20 and 37 °C expression (*error bars* represent S.D. of three independent experiments). *E*, the expression level of full-length Sso7d mutants (c-MYC) was plotted against total surface expression (the HA tag is located between Aga2p and Sso7d) (expression at 20 °C).

Using the charge-neutralized mutant rcSso7d as a scaffold, we constructed yeast display libraries that were randomly mutated at nine positions within three adjacent β-strands (Fig. 1*A*, *magenta*). Because only every other position is randomized, all nine residues are solvent-exposed. To increase the frequency of potential binders in the library, we mimicked the amino acid diversity found in protein-protein interactions (PPIs) using two previously published data sets: (i) the -fold enrichment of amino acids in PPI hot spots (defined as ΔΔ*G* ≥ 2 kcal/mol in alanine scans) over the entire database of alanine mutations that was analyzed in the study by Bogan and Thorn (23) and (ii) the frequency of amino acids in CDR-3 loops of antibody heavy chains (CDR-H3) (24). We excluded amino acids that were infrequent in CDR-H3 and showed low enrichment in PPI hot spots (Fig. 2*A*, *red letters*). In addition, proline was excluded to avoid disruption of the β-strands. This resulted in the library rcSso7d-11, which was designed to contain a mixture of 11 different amino acids (Fig. 2*A*, *black letters*) at equal frequencies (9.1% each) at all nine randomly mutated positions. As a control, we constructed the library rcSso7d-18 containing all amino acids at a frequency of 5.6% each except for proline and cysteine. To control the distribution of amino acids in a precise manner, the randomized oligonucleotides were constructed by trinucleotide synthesis. Finally, both rcSso7d-11 and rcSso7d-18 were constructed as yeast display libraries with 1.4 × 10^9^ transformants each.

We confirmed that the distribution of amino acids in the binding sites matched the designed frequencies very well (Fig. 2*B*). Importantly, both libraries were expressed on the surface of yeast at high levels (Fig. 2, *C* and *D*). This indicates that the vast majority of the mutant proteins in the libraries are well folded and thermally stable as multiple studies have demonstrated that expression levels correlate with thermal stability (25–27). When full-length display, confirmed by the presence of the c-MYC tag, was plotted against total surface expression of the anchor protein Aga2p (using the HA tag), the resulting diagonals were almost indistinguishable between the libraries and Sso7d-WT or rcSso7d (Fig. 2*E*), again confirming the high tolerance to mutation. Only a minor fraction of the libraries displayed truncated proteins (HA-positive, c-MYC-negative), likely due to the introduction of frameshifts and stop codons (data not shown). This high fraction of expressed and screenable diversity is an unusual feature by comparison with single chain Fv (28) or Fn3 (29) libraries displayed on yeast.

##### Selection of Binders against MSA and hEGFR

To test the diversity of binders against different target proteins that can be selected from the libraries rcSso7d-11 and rcSso7d-18, we chose two structurally distinct model antigens: MSA and the extracellular domain of hEGFR. Libraries rcSso7d-11 and rcSso7d-18 were screened separately to compare the properties of resulting clones.

**FIGURE 3.**
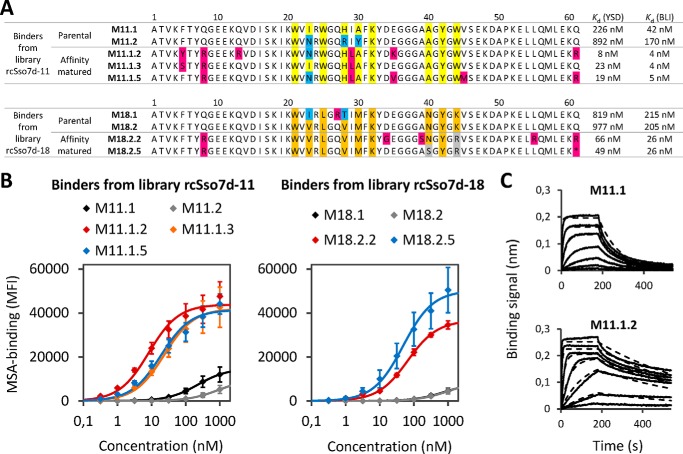
**Analysis of selected MSA binders.** *A*, sequences of selected clones are depicted for libraries rcSso7d-11 (*top*) and rcSso7d-18 (*bottom*). Identical amino acids within one library at the nine randomly mutated positions are depicted in the *same colors*. Framework mutations are highlighted in *magenta*, and mutations in the binding site with respect to the parental clone are in *gray*. The *asterisk* at the last position of M18.2.5 indicates a stop codon. On the *right*, *K_d_* values obtained from yeast surface display (*YSD*) titrations and BLI measurements are shown. *B*, rcSso7d mutants were displayed on the surface of yeast and titrated with MSA. Parental clones are depicted in *black* and *gray*, and affinity-matured binders are depicted in *red*, *orange*, and *blue. Error bars* represent S.D. of three independent experiments. Data were fitted to a 1:1 binding model (*solid lines*) to calculate the *K_d_* values in *A. C*, BLI measurements for M11.1 and M11.1.2. Measured data (*solid lines*) and curves from a global fit 1:1 binding model (*dashed lines*) are represented. *K_d_* values that were obtained from steady state analysis are shown in *A* on the *right* (averages of two independent experiments). *MFI*, mean fluorescence intensity.

MSA selections were started with two rounds of enrichment for binding to MSA-loaded magnetic beads (30). To reduce the frequency of nonspecific binders and streptavidin binders, we also performed three rounds of negative selection against unloaded streptavidin beads. Subsequently, the magnetic bead-enriched libraries were sorted by FACS three times. Both rcSso7d-11 and rcSso7d-18 converged to one sequence family with the two most frequent clones for each library shown in Fig. 3*A* (M11.1 and M11.2 for library rcSso7d-11 and M18.1 and M18.2 for rcSso7d-18). Yeast surface titration of the MSA binders revealed affinities in the high nanomolar range (Fig. 3, *A* and *B*, *black* and *gray curves*), with M11.1 having the highest affinity (226 nm). Next, these four binders were subjected to two rounds of affinity maturation with each round consisting of error-prone PCR (epPCR) and two to three FACS sorts. For each of the two library designs (rcSso7d-11 and rcSso7d-18), the two lead clones were affinity-matured in the same tube, resulting in direct competition between mutants derived from either of the two parental clones. The majority of the resulting clones were variants of M11.1 and M18.2, respectively. The affinity-matured binders are referred to here according to their parental clone. For example, M11.1.2 is a variant of M11.1. MSA titrations of yeast-displayed binders showed 10–30-fold improvements in affinity over the respective parental clones (Fig. 3, *A* and *B*, *colored curves*) with M11.1.2 being the strongest binder with a *K_d_* of 8 nm. Finally, the results from yeast surface titrations were confirmed by biolayer interferometry (BLI) where solubly expressed rcSso7d clones were incubated with MSA-loaded tips. Representative measurements are shown for the parental binder M11.1 and its affinity-matured version M11.1.2, clearly demonstrating an increase in affinity during affinity maturation (Fig. 3*C*). Affinities obtained from BLI experiments were severalfold higher compared with yeast surface titrations, but the relative ranking of affinities was consistent between the two methods. According to BLI, the highest affinities were 4 nm for M11.1.2 and M11.1.3 (Fig. 3*A*).

**FIGURE 4.**
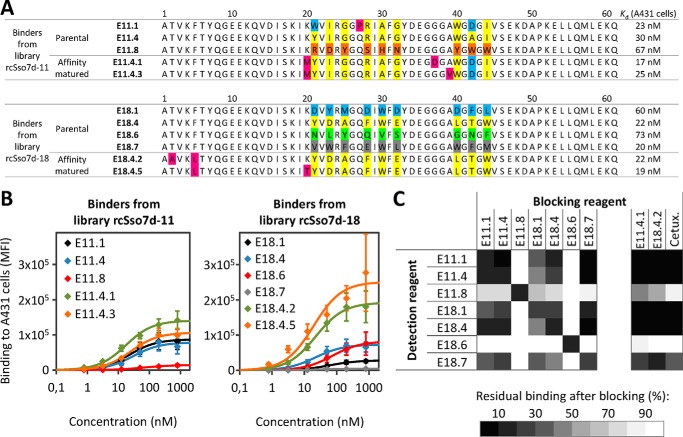
**Analysis of selected hEGFR binders.** Sequences obtained after hEGFR selection are shown for libraries rcSso7d-11 (*top*) and rcSso7d-18 (*bottom*). For each library, the nine randomized positions of sequence families are highlighted with *one color*. Framework mutations are shown in *magenta. B*, A431 cells were titrated with His_6_-SUMO-rcSso7d mutants, and binding was detected with anti-His-Alexa Fluor 647 (*error bars* represent S.D. of three independent experiments). The *lines* show the fits to a 1:1 binding model with *K_d_* values shown in *A. C*, A431 cells were preincubated with 1.5 μm rcSso7d mutants without His tags or with 300 nm cetuximab (*Cetux.*) (blocking reagent) followed by addition of 20 nm His_6_-SUMO-rcSso7d mutants (detection reagent) and detection with an anti-His antibody. Average blocking levels of two independent experiments are shown. *MFI*, mean fluorescence intensity.

For hEGFR selections, a fusion protein consisting of the extracellular domain of hEGFR and human IgG1-Fc (hEGFR-Fc) was used. Similar to MSA selections, both libraries were subjected to two rounds of positive and three rounds of negative magnetic bead selections using hEGFR-Fc-loaded and unloaded beads, respectively, followed by FACS. To avoid enrichment of Fc binders, one FACS round of negative selection against hIgG1-Fc was included. 18–36 clones were sequenced from both libraries after the third, fourth, and fifth FACS rounds. Sequence analysis showed that, in contrast to MSA, several distinct sequence families had emerged (Fig. 4*A*).

To narrow down the number of mutants for further analysis, individual mutants were displayed on the surface of yeast and tested for binding to hEGFR-Fc (data not shown). In addition, these clones were also analyzed for competition with the clinical anti-EGFR antibody cetuximab. Remarkably, all tested mutants except for two (E11.8 and E18.6) were blocked by preincubation of hEGFR-Fc with cetuximab, indicating that the majority of the isolated rcSso7d mutants binds to an epitope overlapping with that of cetuximab (data not shown). Based on single clone analysis, we chose a set of mutants for affinity maturation (parental clones in Fig. 4*A*). Instead of choosing only the binders that showed the highest signal for hEGFR-Fc binding, we also included binders from different clone families, such as the non-cetuximab-competitive binders E11.8 and E18.6. Similar to MSA affinity maturations, for each of the two library designs, one affinity maturation library was constructed starting with a mixture of the parental clones. After two rounds of affinity maturation, binding was significantly improved compared with parental clones (data not shown). To determine monovalent *K_d_* values, the binders were expressed solubly and titrated on human EGFR-positive A431 cells (Fig. 4, *A* and *B*). Binders isolated from the naïve library without affinity maturation already showed monovalent affinities of up to 20 nm. In agreement with yeast display experiments, the binding signal of affinity-matured binders was increased (Fig. 4*B*, *green* and *orange curves*). However, the *K_d_* values were only slightly improved compared with the parental clones (Fig. 4*A*), which may be due to increased repulsion between anionic cell surfaces and the negative charges that accumulated on the binders during affinity maturation.

To investigate whether the isolated hEGFR binders bind to overlapping epitopes, we performed competition experiments on A431 cells. Briefly, cells were preincubated with non-tagged rcSso7d mutants or cetuximab followed by His-tagged binders, which were detected with an anti-His_6_ antibody (Fig. 4*C*). In agreement with yeast display competition experiments, all binders except for E11.8 and E18.6 competed with cetuximab. Moreover, all of these cetuximab-competitive binders also competed with each other for binding to hEGFR, suggesting that they all bind to a common hot spot on hEGFR. Importantly, E11.8 and E18.6 did not compete with each other, indicating that they recognize two distinct epitopes on hEGFR (Fig. 4*C*). Together, these experiments demonstrate that rcSso7d can be engineered for binding to different epitopes on natively folded hEGFR on human cancer cells with high affinities. Moreover, the interaction with the cells is highly specific for EGFR because binding is efficiently blocked by preincubation with either non-tagged rcSso7d-based binders or with the clinically approved antibody cetuximab.

##### Strong Enrichment of Aromatic Residues in Binding Surfaces

To evaluate the distribution of amino acids in the rigid binding surfaces of rcSso7d-based binders, we performed deep sequencing analysis of EGFR-selected rcSso7d-11 and rcSso7d-18 libraries. We chose the EGFR libraries because they contained distinct sequence families (Fig. 4*A*) as opposed to MSA libraries, which converged to one prominent clone family (Fig. 3*A*).

**FIGURE 5.**
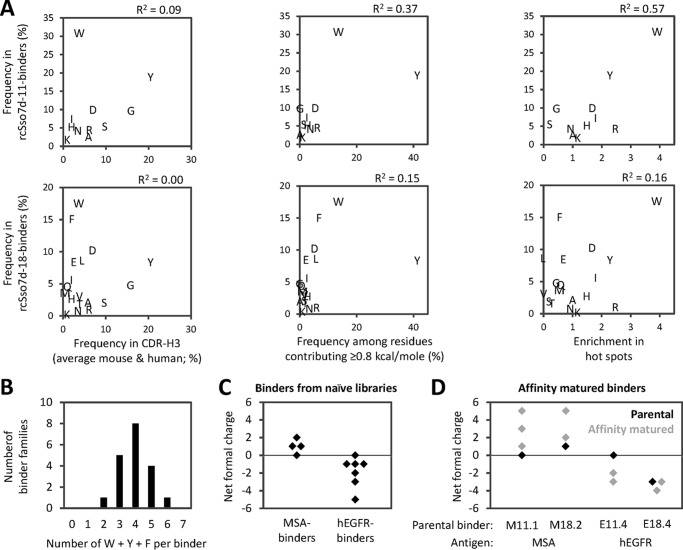
**Strong enrichment of aromatic residues and antigen-dependent adjustments in charge in selected binders.** *A*, hEGFR-selected libraries derived from rcSso7d-11 and rcSso7d-18 were analyzed by deep sequencing. Amino acid frequencies at the nine randomized surface positions in rcSso7d-11-derived binders (*top*) and rcSso7d-18-derived binders (*bottom*) are plotted against the frequencies in CDR-H3 loops (24) (*left*) or among residues that contribute ≥0.8 kcal/mol to binding in a computational alanine scan (31) (*middle*). In addition, amino acid distributions in rcSso7d-11 and rcSso7d-18 clones were compared with experimentally derived enrichments in PPI hot spots (23) (*right*). *B*, number of aromatic residues (Trp + Tyr + Phe) per binder. Reads from deep sequencing were clustered to sequence families, and each family was counted as one binder. *C*, net formal charges of MSA binders (from Fig. 3*A*) and hEGFR binders (from Fig. 4*A*) that were selected from the naïve libraries without affinity maturation. *D*, net formal charges of the parental clones (*black*) and their affinity-matured variants (*gray*) that are shown in Figs. 3*A* and 4*A*. On the *bottom*, the respective parental clone and the antigen are indicated.

All reads from deep sequencing were first grouped into six and 13 sequence families for rcSso7d-11 and rcSso7d-18, respectively. Reads within a family contained similar sequence patterns with only few variations at the nine binding site positions. Subsequently, each family was treated as one clone for further analysis to avoid over-representing the most abundant clones. Finally, the frequency of amino acids in the binding surface of hEGFR binders was analyzed and plotted against that in antibody CDR-H3 loops (24) (Fig. 5*A*, *left*). Surprisingly, there was little to no correlation between the amino acid frequencies in rcSso7d-based binding surfaces and in antibody CDR-H3 loops (*R*^2^ of 0.09 and 0.00 for rcSso7d-11 and rcSso7d-18, respectively). In contrast, the amino acid distribution in rcSso7d-based binders shows similar trends compared with that at antibody hot spots (residues contributing at least 0.8 kcal/mol to the free energy of binding in a computational alanine scan of 227 antibody-antigen complexes (31)) with *R*^2^ values increasing to 0.37 and 0.15 for rcSso7d-11 and rcSso7d-18, respectively (Fig. 5*A*, *middle*). For example, in both antibody hot spots and binding surfaces from library rcSso7d-11, the two most frequent residues are *W* and *Y*. A similar trend was observed for rcSso7d-18-derived binders with the top six amino acids (Trp, Phe, Asp, Leu, Glu, and Tyr) closely resembling the top six in antibody hot spots (Tyr, Trp, Phe, Arg, Leu, and Asp). This agreement with data from a computational alanine scan was confirmed by comparing the frequencies of amino acids in rcSso7d-based binders with experimentally determined enrichments in PPI hot spots (Fig. 5*A*, *right*) (23). Again, the two most frequent residues in rcSso7d-11-derived binders (Trp and Tyr) are the most and third-most enriched in PPI hot spots. Although this correlation was less evident for binders from rcSso7d-18, the most frequent residue in those binders (Trp) was also the most enriched in PPI hot spots. This observation was confirmed by increased *R*^2^ values for both libraries (Fig. 5*A*, *right*) compared with the *R*^2^ values obtained from correlations with overall distributions in antibody CDR-H3 loops (Fig. 5*A*, *left*).

Together, these results demonstrate that, although the amino acid distribution in the small and rigid binding surface of rcSso7d-based binders resembles that at PPI energetic hot spots, it shows little correlation with the distribution in more flexible antibody CDRs, suggesting that the optimal amino acid mixture depends on the rigidity of the paratope. Particularly striking is the high frequency of Trp (being the most frequent amino acid in binders from both rcSso7d-11 and rcSso7d-18) as well as the two other aromatic amino acids, Tyr and Phe. Remarkably, analysis of the sequence families obtained by deep sequencing showed that the vast majority of hEGFR binders contain three to five of these aromatic amino acids in their nine-residue binding surfaces, and no binder contained less than two of these residues (Fig. 5*B*). The MSA binders discussed above (Fig. 3) also contain two to five of these aromatic residues in their binding sites, showing that the high frequency of Trp, Tyr, and Phe residues is consistent among rcSso7d-based binders against both antigens.

**FIGURE 6.**
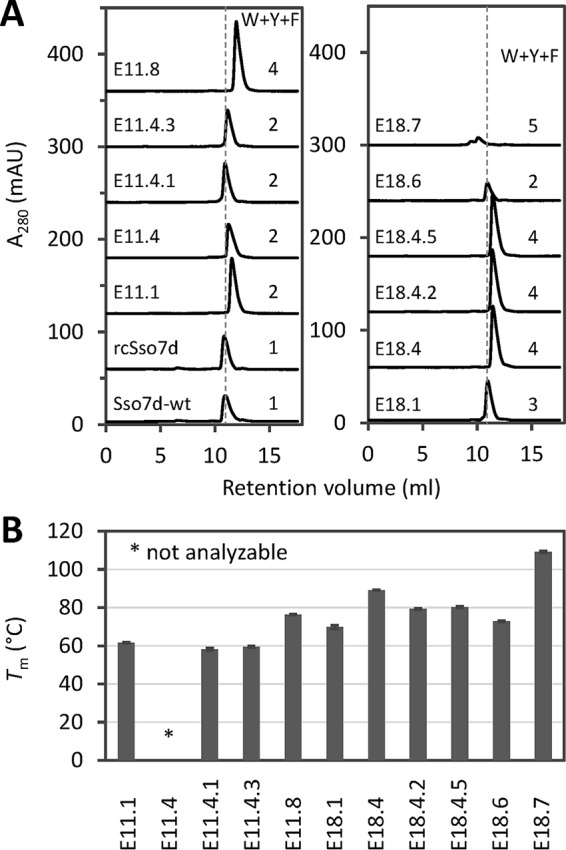
**Selected hEGFR binders are stable and monomeric.** *A*, analysis of rcSso7d mutants by size exclusion chromatography using a TSKgel G2000SWxl column. For comparison, the elution time for Fc-WT is indicated with *gray dashed lines*. The reason for the low signal of E18.6 is the absence of Trp residues in this protein, resulting in low absorbance at 280 nm. The number of Trp, Tyr, and Phe residues in the binding site is shown for each mutant. *B*, *T_m_* values measured by DSC (*error bars* represent S.D. of two to three measurements). The *T_m_* of E11.4 could not be analyzed due to low signal. *mAU*, milli-absorbance units.

To investigate whether the high frequency of aromatic amino acids negatively impacts the biophysical properties of the resulting binders, we analyzed the diverse set of hEGFR binders shown in Fig. 4 by size exclusion chromatography (SEC) and DSC. The number of Trp, Tyr, and Phe residues in the binding sites of these binders range from 2 to 5, closely matching the distribution obtained from deep sequencing. Remarkably, SEC analysis demonstrated that all mutants elute as single peaks that are almost indistinguishable from Sso7d-WT and rcSso7d (Fig. 6*A*), suggesting they are monomeric. Importantly, no aggregation was observed for any of these binders except for E18.7, which contains three Trp and two Phe residues in the binding surface. Moreover, the majority of binders are highly stable with *T_m_* values above 70 °C as shown by DSC analysis (Fig. 6*B*). Only a single sequence family (E11.1, E11.4.1, and E11.4.3), containing two aromatic amino acids, is less stable with *T_m_* values of around 60 °C. Together, these results demonstrate that the nine-residue binding surface tolerates up to four aromatic side chains, yielding highly stable binders with SEC elution profiles very similar to wild-type Sso7d.

##### Charge Adaptation to the Antigen

Finally, to evaluate how the overall charge of the binders evolved during selection, we calculated the net formal charges for all sequences shown in Figs. 3 and 4. We observed a strong antigen-dependent difference in charge. Although there was a trend toward positive charges in MSA binders, hEGFR binders were predominantly negatively charged (Fig. 5*C*). Moreover, comparing the net formal charges of affinity-matured binders with that of their parental clone demonstrated that the antigen-dependent charge difference was further enhanced during affinity maturation with MSA binders becoming more positive and hEGFR binders becoming more negative (Fig. 5*D*). Thus, there was a clearly different requirement for positively or negatively charged residues depending on the charge environment of the antigen that was used during selection as will be discussed below.

## Discussion

In this study, we engineered potential improvements to the hyperstable Sso7d protein in an attempt to make it a better scaffold for molecular recognition. First, we charge-neutralized this highly positively charged DNA-binding protein by deleting the two C-terminal lysines and mutating four lysines to neutral amino acids. The resulting mutant rcSso7d maintained high thermal stability (*T_m_* of 95.5 °C), and its net formal charge was reduced from +7 to +1. Because one Lys and one Arg residue were part of the binding surface that was randomized in the libraries, the final net formal charge of the library scaffold was −1. In addition, our improvement of the Sso7d scaffold also involved flattening the binding surface. To remove the protuberance at position Lys-39 and thereby avoid clashes with interacting antigens, this residue was mutated to the small side chain of alanine.

The reason for charge-neutralizing the protein was twofold. First, it has been shown in numerous studies that positively charged proteins interact nonspecifically with negatively charged cell surfaces (19–21). Consistent with these reports, we observed a strong correlation between the net formal charge of Sso7d mutants and nonspecific binding to HeLa cells with rcSso7d being completely negative for HeLa cell binding. It should be noted that incubation with DNase I partly abolished cell binding, suggesting that the DNA binding properties of Sso7d-WT were at least partly responsible for the observed effect (data not shown). In line with this observation, large amounts of DNA co-purified with Sso7d-WT, an effect that was strongly reduced for rcSso7d and completely abolished for all binders (data not shown).

Second, we hypothesized that the high concentration of positively charged residues around the binding surface would hinder selection of binders against positively charged epitopes, whereas a charge-neutralized scaffold would be able to easily adapt to different charge-environments of different epitopes. Indeed, we observed strong antigen-dependent charge differences in the selected binders. Selection for MSA binding resulted in positively charged binders, whereas hEGFR binders were negatively charged. This difference was further enhanced during affinity maturation. Thus, in the case of MSA selections, the binders adapted to the negatively charged antigen (theoretical isoelectric point (pI) of 5.5) by enriching positively charged amino acids. Interestingly, the extracellular domain of hEGFR is almost neutral in charge, not explaining the trend toward negatively charged hEGFR binders. However, the crystal structure of the antigen-binding fragment (Fab) of cetuximab bound to the extracellular domain of hEGFR shows that the cetuximab epitope on hEGFR involves three positively charged amino acids (Lys-443, Lys-465, and Arg-353) but no negatively charged side chains (32). Moreover, two of these residues on hEGFR (Lys-443 and Lys-465) form salt bridges with Asp-58 and Asp-103 on CDR-H2 and CDR-H3 of cetuximab, respectively. Because competition experiments showed that cetuximab and the majority of hEGFR binders selected in this study have overlapping epitopes, the concentration of positive charges in the epitope of cetuximab may explain the enrichment of negative charges in these hEGFR binders. Together, these results support our initial hypothesis that starting from a charge-neutralized scaffold allows selection of binders against both positively and negatively charged epitopes. It seems unlikely that a library based on wild-type Sso7d, which has a ring of positive charges surrounding the binding surface (Fig. 1*A*), would have yielded high affinity binders against this positively charged epitope on hEGFR.

Because yeast display levels have been shown to correlate with protein fitness, in particular with thermal stability (25–27), we analyzed the yeast surface expression levels of Sso7d-WT and rcSso7d. Although Sso7d-WT was already very efficiently displayed, expression levels of rcSso7d were slightly higher, suggesting that the reduction in charge improved the overall fitness of the protein as a recognition scaffold for library construction. Furthermore, the high stability of rcSso7d, in combination with the rigid binding surface, enabled a high tolerance to mutations as the yeast surface expression profiles of both libraries (rcSso7d-11 and rcSso7d-18) were almost indistinguishable from the single clones Sso7d-WT and rcSso7d. Such a result is unusual for naïve libraries but in line with a previous study that demonstrated that high protein stability improves the tolerance to mutations and protein evolvability (18).

The rcSso7d-11 and rcSso7d-18 libraries yielded high affinity clones for both MSA and hEGFR. After two rounds of affinity maturation, MSA binders with single digit nanomolar affinities were obtained, which is striking given the small binding surface that only contains nine amino acids. Moreover, hEGFR binders against three different epitopes with affinities up to 20 nm were isolated from the naïve libraries without any affinity maturation. Although affinity maturation of these hEGFR binders resulted in increased binding signals when measured on EGFR-positive A431 cells, the improvements in *K_d_* were moderate. One possible explanation for the moderate improvement in affinity could be the enrichment of negative charges during affinity maturation, possibly leading to increased repulsion from the anionic mammalian cell surfaces.

Apart from charge-neutralizing and flattening the binding surface, we also mimicked the amino acid distribution found in natural PPIs. We restricted diversity to a set of 11 amino acids (library rcSso7d-11) that show high enrichment in PPI hot spots (23) and/or high frequency in antibody CDR-H3 loops (24). Proline was excluded to prevent the disruption of the β-strands that form the binding surface. To test whether this library design improves library quality, we also constructed a control library containing all amino acids except for Pro and Cys (library rcSso7d-18). Although the MSA binders from rcSso7d-11 showed about 5-fold higher affinities compared with rcSso7d-18-derived binders (Fig. 3), there were no apparent differences in affinities between rcSso7d-11- and rcSso7d-18-derived hEGFR binders (Fig. 4). Thus, it seems that the limited amino acid diversity in rcSso7d-11 is either equal to or slightly better than the less restricted diversity in rcSso7d-18. Although this demonstrates that the chosen 11 amino acids are indeed important for protein recognition, restricting diversity to these amino acids does not appear to make the library perform significantly better by the metrics examined.

To investigate the amino acid preferences of the small and rigid binding surface of rcSso7d-based binders, the more diverse populations of hEGFR binders were analyzed by deep sequencing. Surprisingly, there was hardly any correlation between the frequency of amino acids in rcSso7d binding surfaces and the distribution in antibody CDR-H3 loops. However, the distribution of amino acids in the binding sites of rcSso7d-based binders closely matched the frequency of residues that strongly contributed to binding (≥0.8 kcal/mol) in a computational alanine scan of 227 antibody-antigen complexes (31). Overall, these results suggest that the design principles for small and rigid binding surfaces are distinct from loop-based recognition domains like antibodies, which require higher flexibility.

Although much effort has been put into exploring the optimal amino acid distribution in the loop-based paratopes of antibodies and Fn3 domains, establishing Tyr, Gly, and Ser as the most important players (33–36), less work has been conducted on binding surfaces located on rigid secondary structures. Looking at the frequencies of individual amino acids in Fig. 5*A*, it is apparent that Tyr is highly frequent in all of the described data sets. In contrast, the other aromatic residues, Trp and Phe, are present only at low frequencies in antibody CDR-H3 loops but strongly enriched among the residues that contribute ≥0.8 kcal/mol to antibody binding in the computational alanine scan as well as in binding surfaces of rcSso7d mutants (Fig. 5*A*). Conversely, the small, flexible amino acids Gly and Ser, which are highly frequent in antibody CDR-H3s, are much less frequent in rcSso7d-based binders. Consistent with the low frequency in hEGFR binders, Ser and Gly are almost never found at antibody hot spots. Thus, it appears that Gly and Ser play an important role in loop-based binding surfaces where flexibility is important for adapting the right antigen binding conformation, but they do not directly contribute to the binding energy as was also suggested by Sidhu and co-workers (34). As a consequence, these two amino acids show low enrichment in the rigid binding surface of rcSso7d where flexibility is not important (or even detrimental). Conversely, the aromatic residues Trp and Phe are less frequent in antibody CDRs but highly abundant in both antibody hot spots and rcSso7d-based binders. In addition to the rigidness of the rcSso7d binding surface, its small size may also have contributed to the high frequency of aromatic residues that was observed. It is likely that a minimum number of hot spot residues are necessary for binding, thus commanding a higher frequency in a smaller paratope. Together, the different patterns of amino acids observed in antibody CDRs and rcSso7d binding surfaces suggest that the rigidity of the binding surface dictates the optimal distribution of amino acids for specific recognition. This hypothesis is in agreement with the observation that the two most frequent amino acids in the ligand-contacting positions of variable lymphocyte receptors (VLRs) are the aromatic amino acids Tyr and Trp (37). Importantly, similar to rcSso7d, the binding surface of VLRs is also composed of β-strands, thus supporting the concept that paratopes located on rigid secondary structures show higher frequencies of aromatic amino acids compared with flexible loops.

It is worth mentioning that the most enriched amino acid in rcSso7d-based binders from both libraries (rcSso7d-11 and rcSso7d-18) was Trp. This strong enrichment for Trp was also observed in computational (31) and experimental (23) alanine scans of PPI hot spots as well as in rigid VLR binding sites (37). However, Trp is represented by only one codon, resulting in low frequencies in the commonly used NNN (1.6%), NNK (3.1%), or NNB (2.1%) library designs. In contrast, in libraries rcSso7d-11 and rcSso7d-18, all 11 and 18 amino acids were incorporated at equal frequencies, resulting in starting frequencies of 9.1 and 5.6% for rcSso7d-11 and rcSso7d-18, respectively. This balanced representation of Trp may have increased the efficiency of binder selection from these libraries.

It should also be noted that Trp, Tyr, and Phe did not show any preference for particular positions within the binding surface. Deep sequencing data showed high frequencies of aromatic amino acids at all nine binding site positions (data not shown).

Importantly, the binders isolated from the rcSso7d libraries were highly stable and monomeric despite some containing up to four aromatic amino acids of their nine-residue binding surface. We hypothesize that the high thermal stability of rcSso7d, paired with the rigidness of its binding surface, prevents the hydrophobic amino acids from interacting nonspecifically and aggregating. Consistent with this hypothesis, it has been reported that disulfide bonds and salt bridges can stabilize aggregation-prone regions in their native conformations, thereby avoiding the structural rearrangements necessary for aggregation and misfolding (38).

Overall, we have demonstrated that libraries based on the charge-neutralized and flattened Sso7d variant rcSso7d yield binders against multiple antigens and epitopes with low nanomolar affinities and beneficial biophysical properties. Recently, rcSso7d-based binders generated in this study were used for the development of highly efficient reagentless biosensors (39), and a binder that was selected from the rcSso7d-11 library was successfully immobilized on cellulose for the development of paper-based diagnostic tests (40). We anticipate that the libraries and binders generated in this study will be useful for many other applications, including tumor targeting (hEGFR) and *in vivo* half-life extension (MSA). Finally, sequence analysis of the isolated binders generated design principles for small and rigid binding surfaces as distinct from the thoroughly explored design principles for antibody CDR loops.

## Experimental Procedures

### 

#### 

##### In Silico Design of Neutralizing Mutations

All calculations were done using an NMR structure of Sso7d (Protein Data Bank code 1SSO) (41). Terminal side chain dihedrals were considered for a 180° flip for all His, Asn, and Gln residues. Hydrogens were added using the HBUILD module in CHARMM using the CHARMM22 parameter force field. Missing side chain atoms were also built using CHARMM.

For *in silico* protein design, a previously described algorithm was used (42). The calculations were divided into two hierarchical components, conformational search and subsequent more detailed energy scoring. The conformational search was done with a rigid backbone, and discrete side chain rotamers for side chains were used. For each specified protein sequence, we exhaustively searched the conformational space using a dead-end elimination search algorithm, which guarantees a global minimum energy conformation for each sequence. If the global minimum energy conformation for a particular sequence was within a certain cutoff compared with the WT, we retained an energy-ordered list of subsequent conformations for the sequences. The top conformations for each sequence were subsequently rescored using a more detailed energy function that accounts for Poisson-Boltzmann continuum electrostatics and continuum solvent van der Waals forces.

##### Expression and Purification of Sso7d Mutants

All Sso7d mutants were expressed as fusion proteins consisting of an N-terminal hexahistidine tag followed by small ubiquitin-like modifier (SUMO) and Sso7d using the pE-SUMO vector (LifeSensors, Malvern, PA). This expression system offers the advantage of generating Sso7d mutants without any attached tags by subsequent digestion with SUMO protease 1 and purification of His-tagged SUMO and SUMO protease. Briefly, Rosetta 2 (DE3) *Escherichia coli* cells were transformed with sequence-verified plasmids. Overnight cultures were diluted 1:100 in terrific broth + kanamycin (50 μg/ml) + chloramphenicol (34 μg/ml) and incubated at 37 °C. When an *A*_600_ of 2 was reached, expression was induced by addition of 1 mm isopropyl β-d-1-thiogalactopyranoside. After overnight expression at 20 °C, the cells were centrifuged, resuspended in sonication buffer (50 mm sodium phosphate, 300 mm NaCl, 3% glycerol, 1% Triton X-100, pH 8.0), sonicated, and centrifuged again. Subsequently, the His_6_-tagged SUMO fusion proteins were purified from the sonication supernatants using TALON metal affinity resin (Clontech). After addition of 10 mm imidazole, the sonicated supernatants were applied onto the resin twice followed by washing with equilibration buffer (50 mm sodium phosphate, 300 mm NaCl, pH 8.0). For the last washing step, 15 mm imidazole was added to reduce nonspecific binding to the resin. Finally, proteins were eluted with elution buffer (equilibration buffer + 250 mm imidazole) and buffer-exchanged to phosphate-buffered saline, pH 7.4 (PBS).

In the case of Sso7d-WT and its point mutants, with which DNA was co-purified, the proteins were further purified by anion exchange chromatography using Q Sepharose Fast Flow resin (GE Healthcare). Samples were buffer-exchanged to 50 mm NaCl, 20 mm Tris-HCl, pH 8.8. Subsequently, they were loaded onto the resin and washed/eluted with increasing concentrations of NaCl (up to 900 mm). The resulting protein samples were buffer-exchanged in PBS again.

For biophysical analysis (DSC and SEC), the SUMO fusion proteins were digested with the protease Ulp1 (SUMO protease 1), resulting in cleavage of the N-terminal His_6_-SUMO tag right before the N terminus of Sso7d. After overnight digestion at 22 °C, the digestion product was purified using TALON metal affinity resin. Digested SUMO, non-digested SUMO fusion proteins, and SUMO protease 1 (all of them containing a hexahistidine tag) bound to the resin and the flow-through, only containing Sso7d, were collected.

##### Preparation of Antigens

Human IgG1-Fc and hEGFR-Fc were cloned into the gWIZ vector (Genlantis, San Diego, CA) with a hexahistidine tag at the C terminus. FreeStyle HEK293F cells (Life Technologies) were cultured in FreeStyle 293 expression medium (Life Technologies) and transiently transfected using polyethylenimine (PEI). After 1 week, the cultures were centrifuged and filtered. 10× PBS was added to the supernatants to achieve a 1× PBS concentration. The resulting solution was applied onto Protein A resin (GenScript, Piscataway, NJ), washed four times with PBS, and finally eluted with 8 ml of 0.1 m glycine, pH 3.5. The proteins were directly eluted into 0.8 ml of 1 m Tris, pH 8.0, to minimize the incubation time at pH 3.5.

Fatty acid-free MSA was purchased from Alpha Diagnostic International (San Antonio, TX). All antigens (hEGFR-Fc, Fc, and MSA) were biotinylated using EZ-Link Sulfo-NHS-LC-Biotin (Life Technologies), and subsequently monomeric protein was purified by size exclusion chromatography using a HiLoad 16/600 Superdex 200 column (GE Healthcare).

##### Construction of Yeast Display Libraries

Two libraries were constructed, differing in the amino acid mixture that was incorporated at the nine randomized positions. To precisely control the frequency of amino acids at those positions, oligonucleotides were constructed by trinucleotide synthesis (Ella Biotech, Martinsried, Germany). For each library, two self-annealing degenerated oligonucleotides were synthesized: ran_fwd_11 and ran_rev_11 for library rcSso7d-11 and ran_fwd_18 and ran_rev_18 for rcSso7d-18. The sequence of ran_fwd_11 and ran_fwd_18 was 5′-GGCGAAGAAAAACAGGTGGATATTAGCAAAATCAAG*XXX*GTG*XXX*CGT*XXX*GGCCAG*XXX*ATT*XXX*TTT*XXX*TATGATGAAGGTGGTGGTGCC-3′, and the sequence of ran_rev_11 and ran_rev_18 was 5′-GCAGTTCTTTCGGTGCATCTTTTTCGCTCAC*XXX*ACC*XXX*ACC*XXX*GGCACCACCACCTTCATCATA-3′; “*XXX*” is a mixture of 11 different codons for library rcSso7d-11 (Ala, Asp, Gly, His, Ile, Lys, Asn, Arg, Ser, Trp, and Tyr at a frequency of 9.1% each) and a mixture of 18 different codons for rcSso7d-18 (codons for all amino acids except for Cys and Pro at a frequency of 5.6% each). The ran_rev oligonucleotides are reverse primers, and therefore the *XXX* codons are antisense codons in those oligonucleotides. The resulting fragment was elongated by PCR twice using the following oligonucleotides: Sso_lib_nest_fwd (5′-GCAACCGTGAAATTCACATACCAAGGCGAAGAAAAACAGGTGGATATTAGCAAAATCAAG-3′) and Sso_lib_nest_rev (5′-TTGCTTTTCCAGCATCTGCAGCAGTTCTTTCGGTGCATCTTTTTCGCTCAC-3′) for the first elongation PCR; CON2_Sso6T8Q_fwd (5′-GGCTCTGGTGGAGGCGGTAGCGGAGGCGGAGGGTCGGCTAGCGCAACCGTGAAATTCACATACCAAGGCG-3′) and CON2_Ssoshort_back (5′-CTATTACAAGTCCTCTTCAGAAATAAGCTTTTGTTCGGATCCTTGCTTTTCCAGCATCTGCAGCAGTTC-3′) for the second elongation PCR. Both sides of the final PCR product contained regions that were homologous to the NheI/BamHI-linearized pCTCON2 vector, facilitating homologous recombination in yeast. The final construct encoded for the fusion protein Aga2p-HA tag-(Gly_4_Ser)_3_ linker-rcSso7d-c-MYC tag. *Saccharomyces cerevisiae* strain EBY100 was transformed by electroporation with the linearized vector and PCR fragments as described previously (43). The two resulting yeast libraries, rcSso7d-11 and rcSso7d-18, contained 1.4 × 10^9^ transformants each.

For affinity maturation, plasmid DNA of various clones was mixed and mutated by epPCR using a 2 μm concentration each of 8-oxo-dGTP and dPTP. 15 epPCR cycles were performed using the primers epSso_fwd (5′-GGCTCTGGTGGAGGCGGTAGCGGAGGCGGAGGGTCGGCTAGC-3′) and epSso_rev (5′-CTATTACAAGTCCTCTTCAGAAATAAGCTTTTGTTCGGATCC-3′). A detailed protocol is provided elsewhere (43). Subsequently, the gel-purified epPCR product was used as the template for a second PCR for amplification of the insert using the same primers that were also used for epPCR. Finally, EBY100 was transformed with NheI/BamHI-linearized pCTCON2 and the insert as described above.

##### Yeast Display Experiments

Yeast display experiments were performed as described previously (43–46). Briefly, for all yeast display experiments, cultures were grown to stationary phase in SD-CAA medium overnight followed by dilution in SD-CAA to an *A*_600_ of 1. After 5 h, cells were centrifuged, and yeast surface expression was induced in SG-CAA overnight. If not stated otherwise, expression was always performed at 20 °C. All subsequent procedures were done in PBSA (PBS + 0.1% bovine serum albumin (BSA)). For all MSA selections and MSA titrations, PBSO (PBS + 0.5% ovalbumin) was used instead of PBSA to avoid blocking potential cross-reactive MSA binders with BSA.

Bead selections were conducted using biotinylated antigen and streptavidin-coated Dynabeads (Life Technologies) as described previously (30, 43). For each selection campaign, two positive (beads with antigen) and three negative bead selections (incubation with bare beads and selection of non-bound cells) were done.

For FACS experiments, washed cells were incubated with biotinylated antigen and mouse anti-c-MYC (clone 9E10) followed by incubation with Streptavidin-Alexa Fluor 647 and goat anti-mouse Alexa Fluor 488 (all reagents from Life Technologies). For hEGFR selections, we alternated between use of biotinylated and non-biotinylated antigen. Non-biotinylated hEGFR-Fc was detected with allophycocyanin (APC)-labeled mouse anti-His_6_ (clone AD1.1.10, Abcam, Cambridge, MA). In this case, the c-MYC tag was detected with chicken anti-c-MYC (Gallus Immunotech, Cary, NC) followed by goat anti-chicken Alexa Fluor 488 (Life Technologies). Cells were either sorted on a FACSAria IIU or analyzed on an Accuri C6 flow cytometer (both from BD Biosciences).

##### Mammalian Cell Binding Experiments

HeLa cells were cultivated in DMEM (ATCC, Manassas, VA) supplemented with 10% heat-inactivated fetal bovine serum (Life Technologies). Cells were harvested by incubating them in trypsin/EDTA (Corning, Manassas, VA). After washing in PBS, cells were incubated with 1 μm His_6_-SUMO-Sso7d mutants in PBS followed by detection of surface-bound proteins with mouse anti-His_6_-APC (clone AD1.1.10, Abcam). During the last washing step before FACS analysis, the cells were stained with propidium iodide (2 μg/ml), and only negative cells (*i.e.* non-necrotic cells) were included in the final analysis.

A431 cells (ATCC) were cultivated in DMEM + 10% heat-inactivated fetal bovine serum and detached using trypsin/EDTA. To determine EGFR affinities, A431 cells were incubated with various concentrations of His_6_-SUMO-Sso7d mutants in PBSA followed by three washing steps with PBSA and detection with anti-penta-His-Alexa Fluor 647 (Qiagen, Venlo, Netherlands). For the competition assay, cells were preincubated with blocking reagent (1.5 μm rcSso7d mutants without the His_6_-SUMO tag or 300 nm cetuximab). After 90 min, 20 nm His_6_-SUMO-rcSso7d mutants was added, resulting in a final concentration of 1.0 μm for the blocking rcSso7d mutants and 200 nm for cetuximab. Finally, the His_6_-SUMO-tagged proteins were detected with anti-penta-His-Alexa Fluor 647.

All mammalian cell experiments were analyzed on an Accuri C6 flow cytometer. To avoid endocytosis, all incubation steps were done at 4 °C.

##### DSC and Analytical SEC

All mutants were analyzed on a VP-DSC capillary cell microcalorimeter (MicroCal, Northampton, MA) at a concentration of 30 μm in PBS. Samples were heated from 20 to 120 °C with a heating rate of 1 °C/min. Buffer baselines were subtracted followed by normalization for protein concentration and fitting with a non-two-state thermal unfolding model. For analytical SEC analysis, 100 μl of a 35 μm solution were loaded onto a TSKgel G2000SWxl column and eluted at a flow rate of 0.7 ml/min using a buffer containing 100 mm phosphate and 150 mm NaCl, pH 6.8.

##### BLI

Samples were analyzed on an Octet RED96 instrument (Pall ForteBio LLC, Menlo Park, CA) using PBS supplemented with 0.1% ovalbumin and 20 μl/liter Tween 20. Biotinylated MSA was immobilized onto streptavidin-coated BLI tips (Pall ForteBio). Subsequently, association was analyzed at various concentrations of SUMO-rcSso7d fusion proteins (1:3 dilutions starting from 1000 to 1.37 nm) followed by measuring dissociation in buffer. Buffer baselines (MSA-loaded tips without addition of binder) were subtracted from the data followed by global fitting to a 1:1 binding model. *K_d_* values were obtained from steady state binding analysis.

##### Deep Sequencing

Plasmids were isolated from yeast libraries that had been selected for hEGFR binding (two rounds of bead selection and five rounds of FACS of which one FACS round was a negative selection against hIgG1-Fc). hEGFR-binding rcSso7d mutant genes were amplified by PCR with primers containing barcode sequences, facilitating pooling of multiple samples in one sequencing run. Subsequently, the pooled sample was analyzed on an SMRT cell using an RSII instrument (Pacific Biosciences, Menlo Park, CA).

Analysis of deep sequencing data was performed in Matlab. For all members of the library, pairwise sequence distances were calculated based on the Gonnet substitution matrix. The results were then hierarchically clustered, and sequence families within the phylogenetic tree were defined as sets of sequences that were connected by a path length less than a specified cutoff. Only sequence families that were present at a frequency of at least 0.4% were considered for further analysis. Subsequently, the amino acid frequencies at the nine positions of the binding surface were calculated for each sequence family followed by normalization of the frequency of each sequence family to 1 to avoid over-representation of amino acids that were present in highly frequent sequence families. Finally, the average amino acid frequency in the binding sites among all sequence families was calculated.

## Author Contributions

M. W. T. and K. D. W. designed the study. M. W. T., J. D. K., E. L., and N. K. M. performed experiments. R. R. S. performed and B. T. designed *in silico* experiments. A. W. T. processed deep sequencing data. M. W. T., R. R. S., N. J. Y., and K. D. W. wrote the paper.

## Footnotes

3 The abbreviations used are:

Fn3: fibronectin type III
rcSso7d: reduced charge Sso7d
MSA: mouse serum albumin
hEGFR: human epidermal growth factor receptor
CDR: complementarity-determining region
DSC: differential scanning calorimetry
PPI: protein-protein interaction
epPCR: error-prone PCR
BLI: biolayer interferometry
EGFR: EGF receptor
SEC: size exclusion chromatography
VLR: variable lymphocyte receptor
SUMO: small ubiquitin-like modifier
8-oxo-dGTP: 8-oxo-2′-deoxyguanosine 5′-triphosphate
dPTP: 2′-deoxy-P-nucleoside 5′-triphosphate
APC: allophycocyanin
SD-CAA: 20 g/L D-glucose, 6.7 g/L yeast nitrogen base, 5 g/L casamino acids, 7.4 g/L citric acid monohydrate, 10.4 g/L sodium citrate, pH 4.5
SG-CAA: 18 g/L galactose, 2 g/L D-glucose, 6.7 g/L yeast nitrogen base, 5 g/L casamino acids, 5.4 g/L Na2HPO4, 8.6 g/L NaH2PO4 monohydrate, pH 6.0.
